# Bilateral anterior tibial artery pseudoaneurysms triggered by minor blunt trauma as the initial presentation of vascular Ehlers-Danlos syndrome with a whole-exon deletion

**DOI:** 10.1016/j.jvscit.2026.102490

**Published:** 2026-08-31

**Authors:** Junya Yokoyama, Yuma Aoki, Kazuto Arita, Yuhei Komoda, Yukitoshi Shirakawa, Takashi Ozaki, Masayuki Sakaki

**Affiliations:** a Department of Cardiovascular Surgery, Kinan Hospital, Wakayama, Japan; b Department of Pathology, Kinan Hospital, Wakayama, Japan

**Keywords:** Anterior tibial artery, Hybrid management, Pseudoaneurysm, Vascular Ehlers-Danlos syndrome, Whole-exon deletion

## Abstract

Bilateral lower-limb arterial lesions after minor trauma are rare and suggestive of vascular Ehlers-Danlos syndrome (vEDS). A 40-year-old man developed bilateral anterior tibial artery pseudoaneurysms after being kicked. The right limb, complicated by compartment syndrome, was treated by anterior tibial artery ligation with aneurysm incision and hematoma evacuation; the left, by coil embolization. COL3A1 sequencing was negative, but multiplex ligation-dependent probe amplification revealed a monoallelic whole-exon deletion, confirming vEDS. Hybrid management is safe in vEDS, and copy-number analysis is essential when sequencing is negative, but suspicion persists.

**Article Highlights**

• Bilateral anterior tibial artery pseudoaneurysms may be the first sign of vascular Ehlers-Danlos syndrome.
• Hybrid open and endovascular treatment achieved successful outcomes.
• Open ligation enabled hematoma decompression in the symptomatic limb.
• Multiplex ligation-dependent probe amplification detected a COL3A1 deletion missed by standard sequencing.
• Advanced genomic testing is crucial when vascular Ehlers-Danlos syndrome is strongly suspected.

Traumatic pseudoaneurysms of the anterior tibial artery (ATA) are usually unilateral; bilateral lesions are exceptionally rare and should prompt evaluation for an underlying vasculopathy.^1^ Vascular Ehlers-Danlos syndrome (vEDS), caused by pathogenic COL3A1 variants, predisposes to arterial dissection, aneurysm, and rupture after minor trauma.^2^ We report bilateral ATA pseudoaneurysms triggered by minor blunt trauma as the initial manifestation of vEDS because of a monoallelic whole-exon COL3A1 deletion, managed by a tailored hybrid strategy.

## Case report

A 40-year-old man with cerebral palsy and severe communication limitations presented with progressive right lower-leg pain and gait disturbance. Two months earlier, he had been kicked on both legs. Left-leg swelling subsided after rest, local cooling, and oral nonsteroidal anti-inflammatory drugs. Right-leg swelling developed 1 month later, was initially treated as cellulitis, and progressed. Despite lifelong joint hyperextensibility and clubfoot deformity (Fig 1, *A* and *B*), he had not been evaluated for a connective tissue disorder. He had no previous vascular event or family history of arterial rupture, or organ rupture, or premature sudden death.

**Fig 1:**
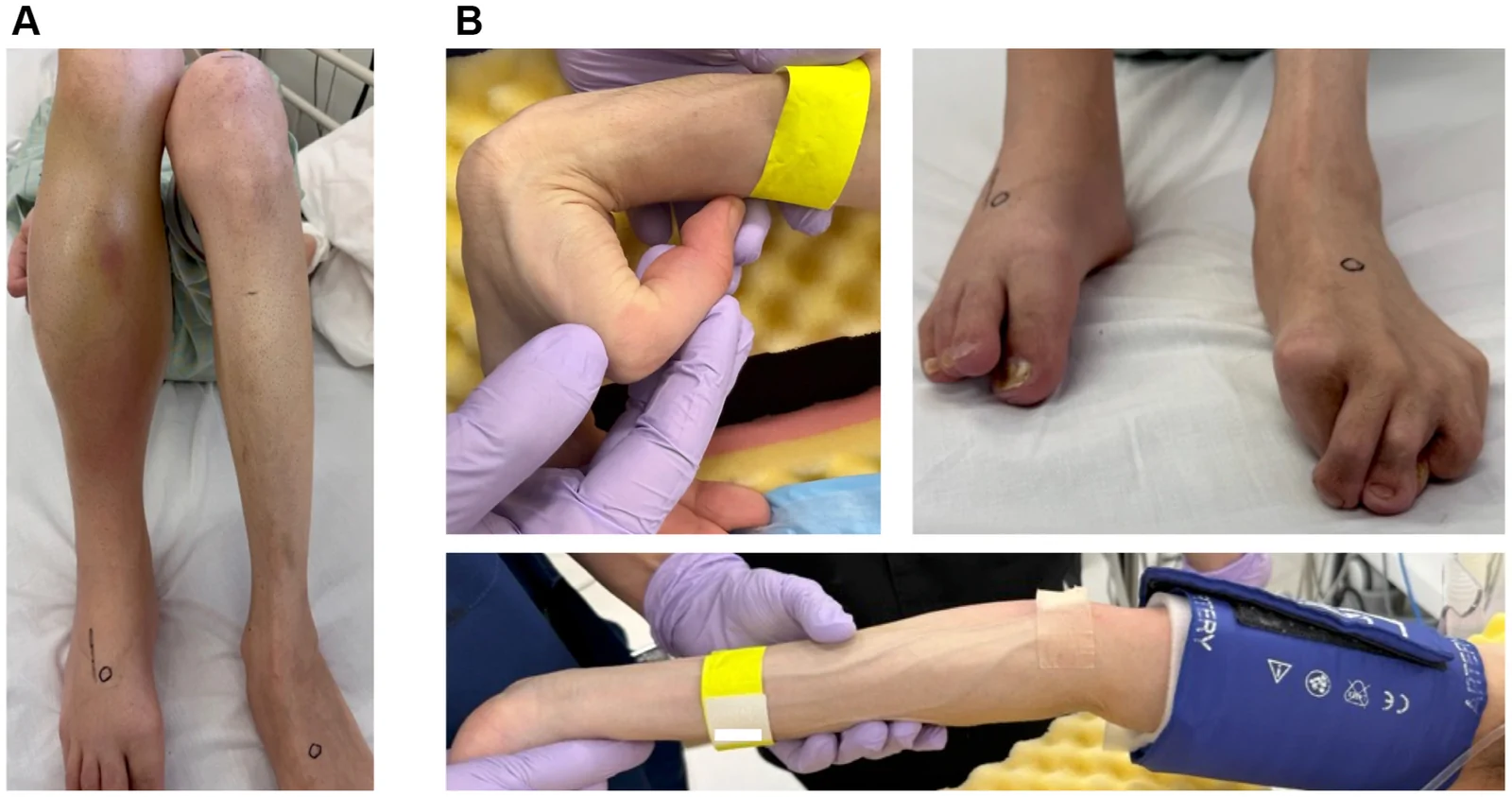
Clinical presentation. **A,** Gross appearance of the lower extremities showing pronounced swelling and ecchymosis on the right lower leg, with an unremarkable contralateral side. **B,** Characteristic physical features of Ehlers-Danlos syndrome (EDS), including wrist and elbow hyperextension and clubfoot deformity.

The right anterior compartment was tense and markedly painful, and distal pulses were diminished. Pain on passive toe extension and reduced active dorsiflexion were present, and compartment syndrome was diagnosed clinically. Intracompartmental pressure was not measured because the clinical findings were considered diagnostic, and cerebral palsy precluded reliable sensory testing. The syndrome had evolved over approximately 1 month, consistent with progressive pseudoaneurysm expansion rather than a single acute ischemic event.

Head-to-pelvis computed tomography (CT) angiography, including the aorta and major branches, together with dedicated CT angiography of the lower extremities, was performed. The former showed no dissection, aneurysm, or other arterial abnormality in the aorta or its branches, with extensive bilateral pulmonary bullae as the only additional finding supporting suspicion of EDS; the latter demonstrated a right ATA pseudoaneurysm with a large hematoma and a smaller left ATA pseudoaneurysm (Fig 2, *A*). Duplex ultrasound confirmed a 27-mm right ATA pseudoaneurysm with turbulent flow (Fig 2, *B*).

**Fig 2:**
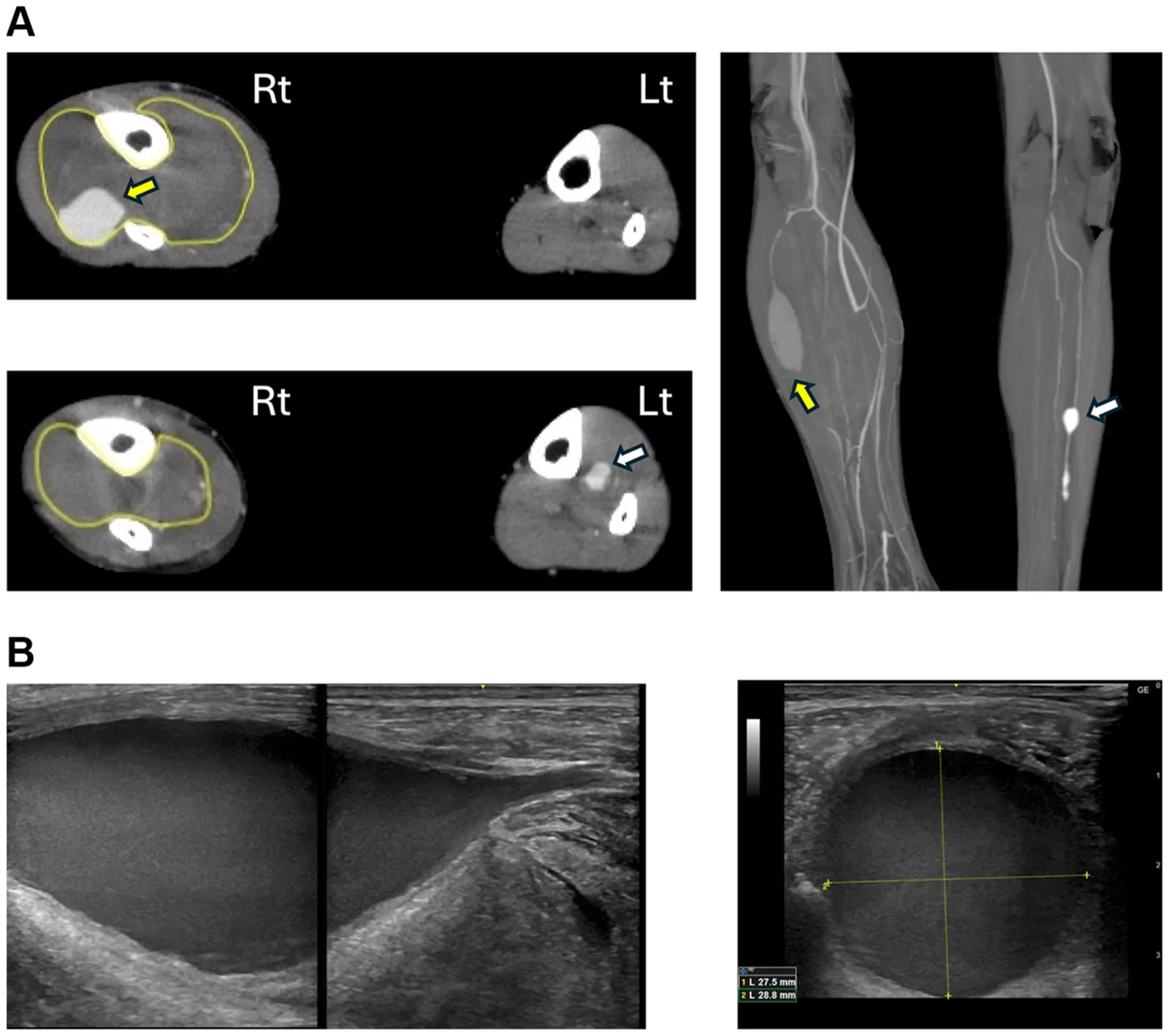
Preoperative imaging. **A,** Computed tomography (CT) angiography demonstrating a large right anterior tibial artery (ATA) pseudoaneurysm (*yellow arrow*) with an extensive hematoma (*yellow box*), and a smaller left ATA pseudoaneurysm (*white arrow*), which was not associated with symptoms at presentation. **B,** Duplex ultrasound confirming the right ATA pseudoaneurysm measuring 27 mm in diameter with turbulent flow. *Lt*, Left; *Rt*, right.

The right ATA was ligated without reconstruction. After ultrasound-guided localization, the proximal and distal ATA segments were exposed through a longitudinal incision (Fig 3, *A*). The deep peroneal nerve, adherent to the pseudoaneurysm wall, was carefully preserved (Fig 3, *B*). After ligation, the pseudoaneurysm was opened, the hematoma evacuated, and an anterior compartment fasciotomy performed through the same incision (Fig 3, *C* and *D*). The other compartments were soft and were not opened. The deep fascia was left unsutured, and the skin was closed primarily. The wound healed without infection, necrosis, or dehiscence. Victoria blue-hematoxylin and eosin staining of the excised aneurysm wall showed attenuation and loss of the elastic fibers of the media (Fig 4, *A*), and Alcian blue staining showed deposition of mucopolysaccharide within the media (Fig 4, *B*), indicating severe medial damage, though these findings are not pathognomonic of vEDS.

**Fig 3:**
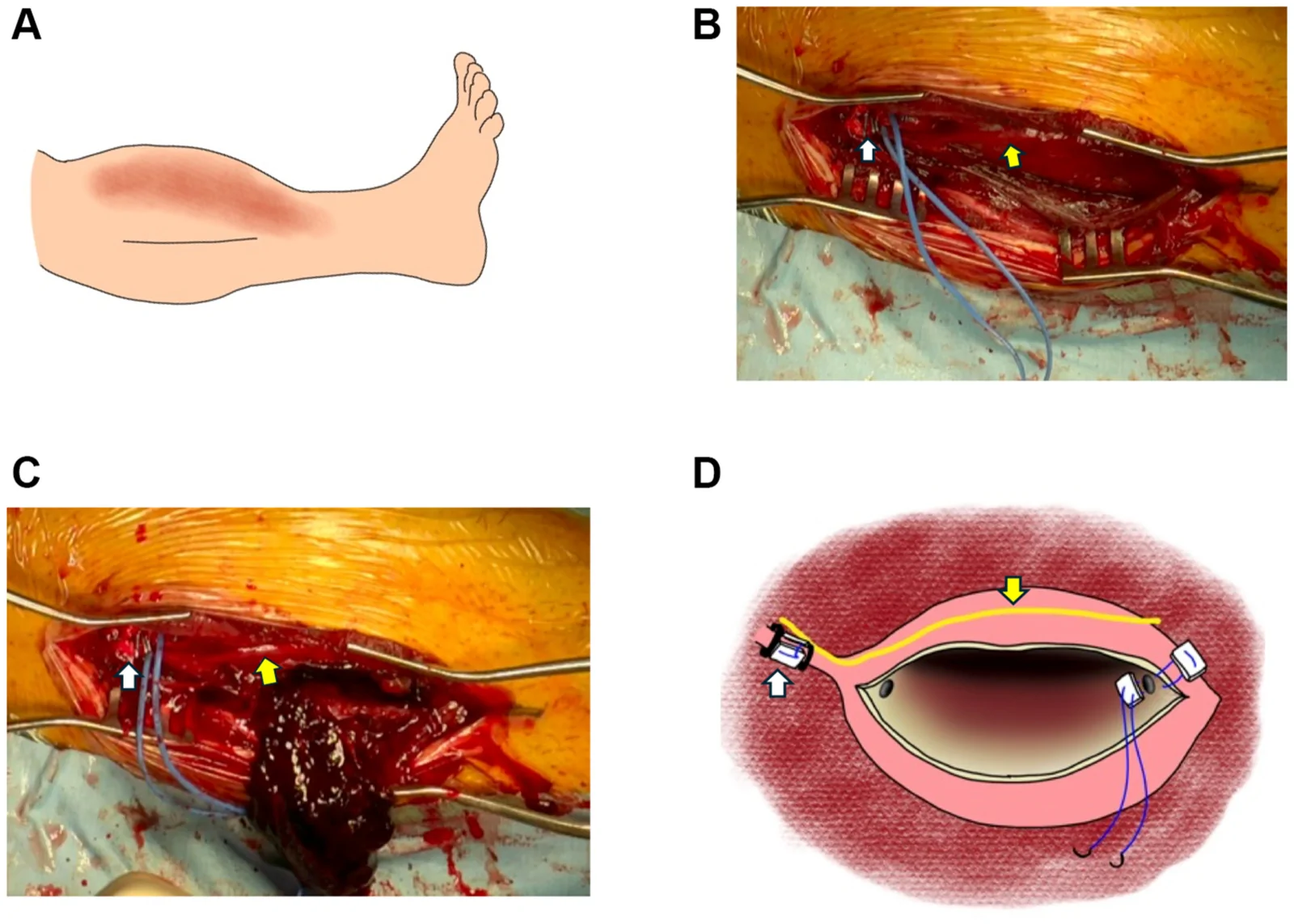
Intraoperative findings of the right lower extremity. **A,** Ultrasound-guided localization to determine the longitudinal incision line. **B,** Careful dissection showing the deep peroneal nerve adherent to the pseudoaneurysm wall (*yellow arrow*) and proximal control of the inflow vessel (*white arrow*). **C,** Incision of the aneurysm wall, evacuation of the hematoma, and fasciotomy of the anterior compartment (*yellow arrow*, deep peroneal nerve; *white arrow*, inflow vessel). **D,** Final operative field showing successful lesion exclusion by double ligation; the deep fascia was left unsutured, and only the skin was closed (*yellow arrow*, deep peroneal nerve; *white arrow*, inflow vessel).

**Fig 4:**
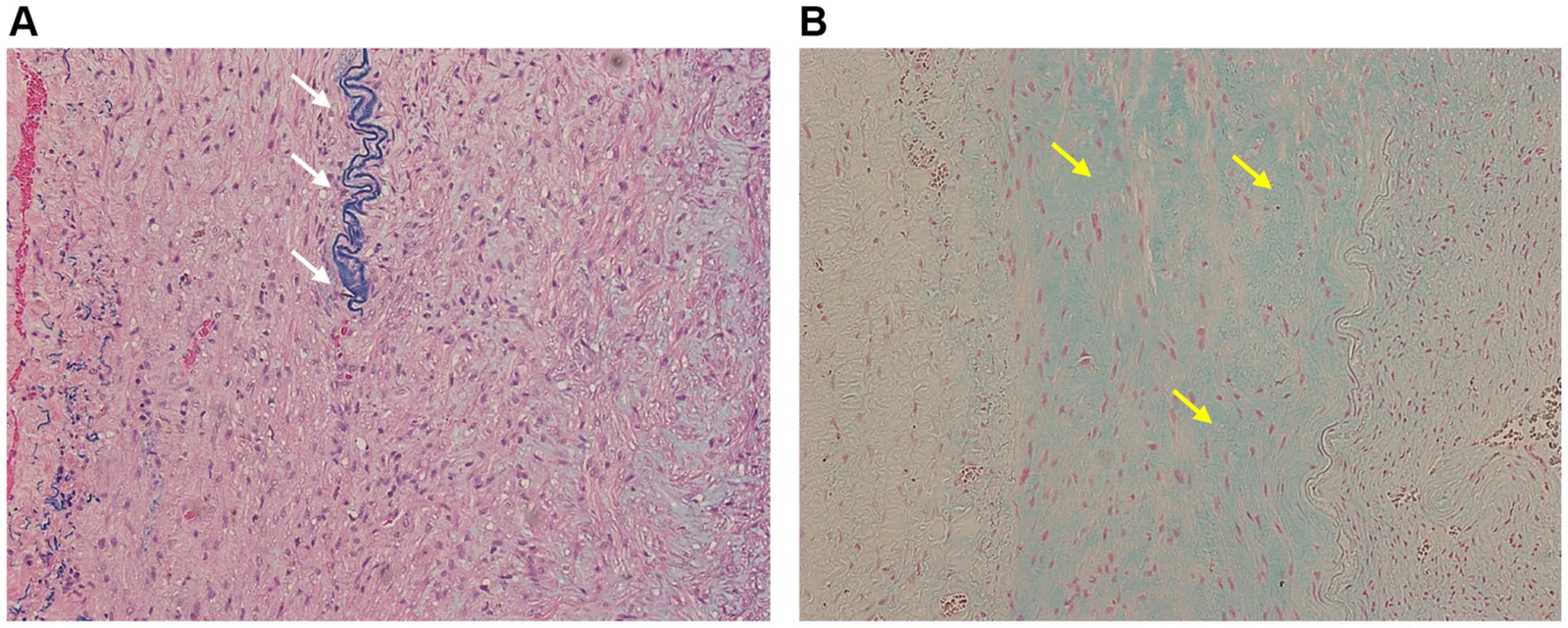
Histopathology of the resected right anterior tibial artery (ATA) aneurysm wall. **A,** Victoria blue-hematoxylin and eosin staining (original magnification ×200), showing attenuation and loss of the elastic fibers (*white arrow*) of the media. **B,** Alcian blue staining (original magnification ×200), showing deposition of mucopolysaccharide (*yellow arrow*) within the media. Together, these appearances indicate severe medial damage, although they are not pathognomonic of vascular Ehlers-Danlos syndrome (vEDS).

One week later, the left ATA was treated endovascularly. An antegrade left common femoral puncture was performed, and a 5F sheath was inserted. A 4F straight catheter was advanced below the knee, and a microcatheter was navigated into the ATA. Angiography confirmed two synchronous pseudoaneurysms (Fig 5, *A*). The sacs and adjacent inflow and outflow segments were embolized with four EMBOLD fibered detachable coils and one Interlock-2D detachable coil (Boston Scientific), completely occluding the affected segment (Fig 5, *B*). No closure device was used; hemostasis was achieved by gentle manual compression and bed rest. Follow-up CT showed no access-site complication.

**Fig 5:**
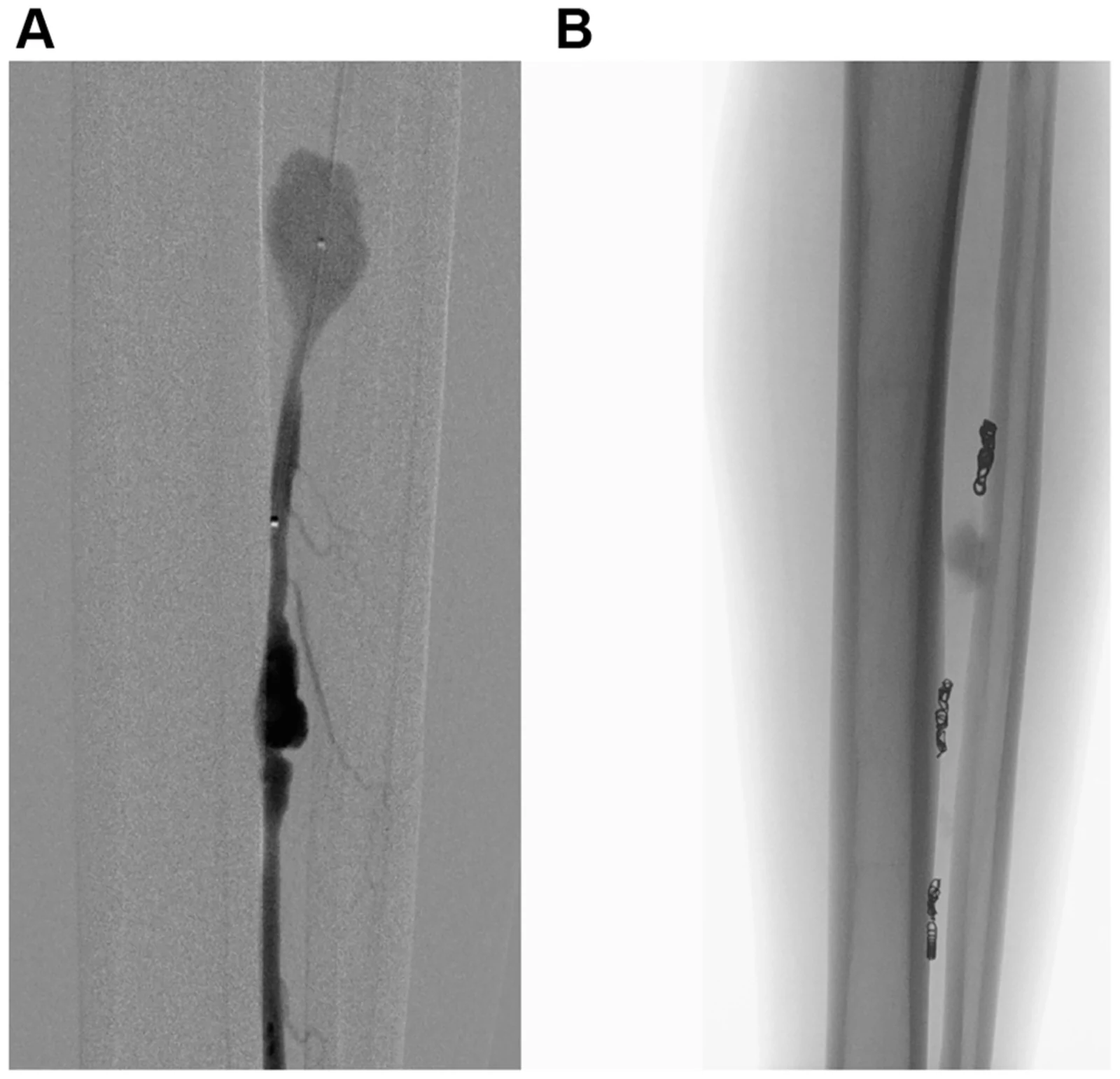
Endovascular intervention for the left lower extremity. **A,** Digital subtraction angiography revealing two synchronous pseudoaneurysms along the left anterior tibial artery (ATA). **B,** Postprocedural angiography demonstrating complete exclusion of both lesions following transcatheter coil embolization. The two panels are displayed at different magnifications: **(A)** shows the overall course of the left ATA, whereas **(B)** is a magnified view of the embolized segment.

Recovery was uneventful, and the patient was discharged on postoperative day 10 with mild residual gait disturbance. Foot perfusion was maintained through the posterior tibial and peroneal arteries; the postoperative ankle-brachial index was 0.92 on the right and 0.94 on the left.

*COL3A1* sequencing detected no point mutation or small insertion/deletion. Because suspicion remained high, copy-number analyses using next-generation sequencing and multiplex ligation-dependent probe amplification (MLPA) identified a monoallelic whole-exon deletion, confirming vEDS. Genetic counseling was provided to the patient and principally to his parents; he had no siblings. Surveillance comprised noncontrast CT every 6 months, together with strict blood pressure control. At 12 months, he remained asymptomatic, with no new or enlarging aneurysm.

Written publication consent was obtained from the patient's parents, his legal representatives; the patient provided assent to the extent permitted by his communication abilities.

## Discussion

The lesions were unlikely to be purely traumatic. Silent aneurysmal degeneration of both ATAs had probably preceded the injury because of vEDS. Trauma may have precipitated right-sided expansion and rupture while unmasking the left-sided lesions; the left-leg swelling had resolved spontaneously. Angiography showed two separate saccular lesions rather than a single focal disruption, a pattern difficult to attribute to one blunt impact. Thus, the vasculopathy rather than the force of injury likely determined the outcome. The bilateral distribution illustrates the multifocal arterial vulnerability of vEDS, in which minor trauma may cause dissection, aneurysm, or rupture.^3^^,^^4^ Whether the lesions were true aneurysms or pseudoaneurysms does not alter the underlying vascular risk.

Standard sequencing may miss copy number variants, which account for approximately 5% of vEDS.^5^^,^^6^ Histologic findings can support the diagnosis but are not specific. When clinical suspicion persists despite negative sequencing, deletion/duplication analysis such as MLPA should be considered. In this patient, MLPA alone established the diagnosis and informed lifelong management, including strict blood pressure control, noninvasive surveillance with duplex ultrasound, noncontrast CT, or magnetic resonance angiography, and avoidance of unnecessary catheter angiography.^4^^,^^7^ Genetic counseling should be offered to the patient and first-degree relatives once the diagnosis is confirmed.

Ligation without reconstruction was selected for the right limb. In vEDS, arterial suturing is technically demanding and carries substantial risks of anastomotic disruption, hemorrhage, and late pseudoaneurysm formation.^7^^,^^8^ However, reconstruction should not be categorically avoided. Experienced surgeons have reconstructed arteries successfully in vEDS,^7^^,^^8^ and reconstruction remains appropriate when vessel sacrifice would threaten life or limb. In this patient, posterior tibial and peroneal artery perfusion permitted ATA sacrifice and immediate compartment decompression.

Intervention on an asymptomatic limb required separate justification. Resolution of symptoms did not indicate resolution of the lesion; patent pseudoaneurysms remained evident on CT and angiography. The right-sided lesion had progressed rapidly after the same minor injury, and anterior-compartment rupture could cause compartment syndrome rather than self-tamponade. This was particularly hazardous in a man who cannot reliably report symptoms. Surveillance would nonetheless have been defensible, as the threshold for prophylactic treatment of infrapopliteal vEDS lesions remains undefined.

Endovascular treatment in vEDS requires caution because catheter manipulation can cause puncture-site disruption, remote dissection, or perforation.^8^ A covered stent has been used successfully in the ATA^9^ but carries risks of edge dissection and neopseudoaneurysm.^3^^,^^10^ Microcatheter coil embolization^11^ excluded the lesions without another open operation, and collateral foot perfusion permitted sacrifice of the vessel.

This report is limited by the fact that compartment pressure was not measured and by short follow-up. Nevertheless, the findings indicated that urgent open decompression and ligation followed by elective coil embolization achieved pain relief, prevented contralateral rupture, and allowed discharge within 10 days, and it may be a safe strategy for bilateral lower-limb arterial lesions complicating vEDS.

## Funding

None.

## Declaration of generative AI and AI-assisted technologies in the writing process

During the preparation of this work, the authors used a generative artificial intelligence tool to assist with language editing (Claude, Anthropic, PBC) and a commercial artificial intelligence-assisted editing service (Editage) for language polishing. After using these tools, the authors reviewed and edited the content as needed and take full responsibility for the content of the publication.

## Disclosures

None.
